# Impact of intracoronary adenosine on the no-reflow phenomenon: A randomized, triple-blind, placebo-controlled clinical trial

**DOI:** 10.1016/j.ahjo.2025.100650

**Published:** 2025-10-20

**Authors:** Fatemeh Baharvand, Mohammadreza Aghajankhah, Shiva Parvaneh, Bahareh Gholami Chaboki, Francesca Maria Di Muro

**Affiliations:** a Department of Cardiology, Healthy Heart Research Center, Heshmat Hospital, School of Medicine, Guilan University of Medical Sciences, Rasht, Iran; b Cardiovascular Disease Research Center, Department of Cardiology, Heshmat Hospital, School of Medicine, Guilan University of Medical Sciences, Rasht, Iran; c The Zena and Michael A. Wiener Cardiovascular Institute, Icahn School of Medicine at Mount Sinai, New York, NY, USA

**Keywords:** Intracoronary adenosine, No-reflow phenomenon, TIMI flow grade, Primary PCI

## Abstract

**Introduction:**

The no-reflow phenomenon occurs in 5 % to 50 % of patients with ST-elevation myocardial infarction (STEMI) during primary percutaneous coronary intervention (PPCI), leading to suboptimal myocardial reperfusion and poor outcomes. Although intracoronary adenosine has shown benefits in studies, its use remains controversial. This analysis aims to evaluate the impact of intracoronary adenosine administration on preventing NRP during PPCI.

**Methods:**

In this randomized, triple-blind, placebo-controlled trial, 240 STEMI patients undergoing PPCI were divided into two cohorts, one receiving a bolus dose of intracoronary adenosine and the other receiving 5 cc of saline as a placebo before stenting. The primary endpoint was the incidence of NRP measured by Thrombolysis in Myocardial Infarction flow grade and frame count. Secondary endpoints included ST-segment resolution after 90 min, left ventricular ejection fraction, and major adverse cardiac events after 40 days.

**Results:**

Among 240 STEMI patients, adenosine did not significantly reduce angiographic no-reflow compared with placebo (TIMI flow grade ≤ 2: 15 % vs. 19.2 %, *p* = 0.391). However, adenosine significantly improved left ventricular recovery at 40 days (ΔLVEF: 13.8 ± 7.4 % vs. 12.1 ± 8.4 %, *p* = 0.043). Multivariable analysis identified diabetes, active smoking, and lower eGFR as independent predictors of no-reflow, while adenosine independently enhanced LVEF recovery.

**Conclusion:**

Prophylactic intracoronary adenosine did not significantly reduce angiographic no-reflow in STEMI patients undergoing primary PCI but was associated with greater left ventricular functional recovery at 40 days. These findings suggest a cardioprotective effect of adenosine on the microvasculature and myocardial tissue, supporting its potential role as an adjunctive therapy in STEMI management.

## 1 Introduction

Current European Society of Cardiology guidelines recommend primary percutaneous coronary intervention (PCI) as the preferred reperfusion strategy for patients with ST-elevation myocardial infarction (STEMI). The greatest benefit is achieved when PCI is performed within the first 2 h of symptom onset and remains effective within the initial 12-h window. Compared to fibrinolytic therapy, primary PCI significantly reduces mortality, reinfarction, and stroke [1]. Despite its benefits, primary PCI carries several acute procedural risks that may negatively influence outcomes. These include coronary artery dissection, vessel perforation, stent thrombosis, and the no-reflow phenomenon (NRP), a particularly serious complication. NRP is defined as inadequate myocardial perfusion despite successful restoration of epicardial coronary artery patency. It occurs in approximately 10–30 % of STEMI patients undergoing primary PCI and is typically identified by a post-procedural Thrombolysis in Myocardial Infarction (TIMI) flow grade of 0 to 2 [2]. NRP is an independent predictor of adverse clinical outcomes, including larger infarct size, reduced left ventricular ejection fraction (LVEF), increased incidence of heart failure, and higher mortality. The risk is even greater in patients who develop cardiogenic shock, which remains the leading cause of in-hospital death among STEMI patients [2,3].

The pathophysiology of NRP mainly results from microvascular injury and dysfunction. Although the exact mechanisms can vary based on patient characteristics and infarct size, key factors commonly involved include endothelial damage, distal embolization, oxidative stress, and inflammation. These processes impair the microvascular circulation despite successful reopening of the epicardial artery [4]. To date, no universally effective treatment strategy has been established [5].

Among various pharmacologic approaches, intracoronary administration of adenosine has shown promising effects due to its vasodilatory, anti-inflammatory, antiplatelet, and antioxidative properties. These include the reduction of calcium overload and reactive oxygen species generation [[6], [7], [8], [9]]. However, prior studies have yielded inconsistent results, likely due to heterogeneity in trial design, patient selection, and dosing protocols, leaving the optimal strategy for NRP prevention unclear [9].

This study aims to assess whether a single intracoronary bolus of adenosine administered before stent deployment can reduce the incidence of no-reflow and improve clinical outcomes in STEMI patients undergoing primary PCI.

## 2 Methods

### 2.1 Trial design

**Fig. 1:**
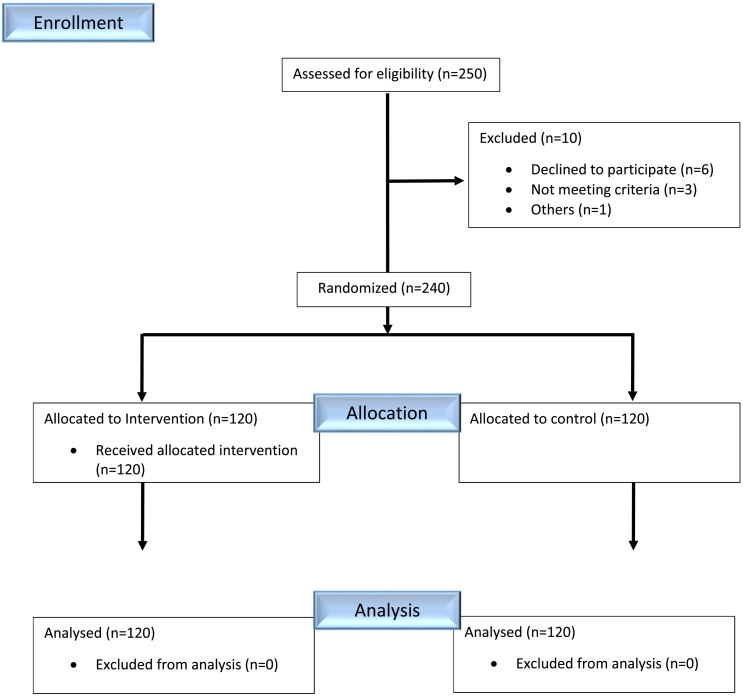
Flowchart of patient enrollment and study design. This flow diagram outlines the screening, randomization, treatment allocation, follow-up, and analysis phases of the clinical trial. A total of 250 patients with STEMI were assessed for eligibility. After applying inclusion and exclusion criteria, 240 patients were randomized into two groups: intracoronary adenosine (*n* = 120) and placebo (n = 120). Follow-up was completed, and all patients were included in the final analysis.

This single-center, triple-blind, randomized, placebo-controlled clinical trial was conducted at Heshmat Heart Center in Rasht, Iran, from May 2023 to June 2024. A total of 250 patients diagnosed with STEMI and eligible for PPCI were enrolled. Inclusion criteria were age ≥18 years and a confirmed STEMI diagnosis in the emergency department. Exclusion criteria included cardiogenic shock, complete atrioventricular (AV) block, severe renal failure (serum creatinine >3 mg/dL), need for emergency coronary artery bypass grafting (CABG), or refusal to participate. The flow chart of the study is summarized in Fig. 1.

### 2.2 Study procedures

After obtaining informed consent, patients were randomized in a 1:1 ratio to either the intervention group (*n* = 120), which received a single intracoronary bolus of adenosine (Adenorytm®, Vianex, Greece) via the guiding catheter immediately before stent deployment. The adenosine dosing protocol was adopted from Mohammad Sadeghian et al. [10], using 200 μg for right coronary arteries and 400 μg for left coronary arteries, administered as a bolus through the guiding catheter immediately prior to stent placement.

Demographic and clinical data, including age, sex, smoking status, and comorbidities (diabetes, hyperlipidemia, hypertension), were extracted from medical records. Serum creatinine levels were used to estimate glomerular filtration rate (GFR) via the MDRD formula. Baseline LVEF was assessed by transthoracic echocardiography. Pre-procedural echocardiography was performed when feasible and did not result in any delay to reperfusion therapy. In patients with a classic clinical presentation and electrocardiographic findings consistent with STEMI, priority was given to immediate primary PCI, and echocardiographic assessment was postponed until after revascularization to avoid unnecessary delay in achieving timely reperfusion.

All patients received standard dual antiplatelet therapy (ticagrelor 180 mg and aspirin 325 mg) before undergoing coronary angiography via femoral or radial access. A single experienced interventional cardiologist performed all procedures to maintain consistency. Intravenous unfractionated heparin (70 IU/kg) was administered before PCI. Use of adjunctive therapies (e.g., thrombus aspiration, glycoprotein IIb/IIIa inhibitors, balloon dilatation) was left to operator discretion.

Post-procedural management followed guideline-directed medical therapy, including statins, beta-blockers, renin-angiotensin system inhibitors, and continued dual antiplatelet therapy. At 40 days, all patients underwent echocardiography to evaluate LVEF. Assessment of major adverse cardiac events (MACE), including cardiovascular death, non-fatal myocardial infarction, or stroke, was performed through outpatient clinic visits or structured telephone interviews.

All patients were continuously monitored with ECG and hemodynamic assessment during and after intracoronary adenosine administration. Any adverse events, including bradyarrhythmia, hypotension, or bronchospasm, were prospectively recorded.

### 2.3 Study endpoints

Our primary endpoint was the occurrence of NRP, assessed using TIMI flow grade and TIMI frame count. Our secondary endpoints included ST-segment resolution (STR), measured 90 min after the procedure, as well as changes in LVEF and the incidence of major adverse cardiac events (MACE) 40 days post-intervention.

The *TIMI Flow Grade* was determined by visually inspecting angiographic images, where the interventional cardiologist evaluated the extent of distal coronary artery filling after the intervention. This angiographic measure of coronary blood flow was categorized as follows:

• Grade 0: No antegrade flow beyond the site of obstruction.
• Grade 1: Minimal flow, with incomplete filling of the distal coronary bed.
• Grade 2: Partial flow, with incomplete but visible filling of the distal coronary bed.
• Grade 3: Complete flow, with normal filling of the distal coronary artery.

NRP was diagnosed if the TIMI Flow Grade is either Grade 0 or Grade 1, indicating inadequate blood flow after intervention.

Conversely, the *TIMI Frame Count* (TFC) measures the speed of blood flow in the coronary artery by counting the number of frames in angiographic images needed for the contrast agent to travel from the point of obstruction to the distal section of the coronary artery. For the Left Anterior Descending (LAD) artery, which is longer than other coronary arteries, we calculated the corrected TIMI frame count (CTFC) by dividing the TFC by 1.7.

The normal values for TFC and CTFC for each coronary territory are as follows:

• LAD: Normal TFC is 36 ± 3 frames; corrected TFC is 20 ± 3 frames.
• Left Circumflex artery (LCX): Normal TFC is 22 ± 4 frames.
• Right Coronary artery (RCA): Normal TFC is 20 ± 3 frames.

No-reflow was diagnosed if the TFC exceeds the normal reference values for the respective artery, or if the CTFC is significantly elevated compared to normal values (e.g., CTFC >36 for LAD).

*T-segment resolution* was measured by comparing the cumulative ST-segment elevation before and 90 min after the procedure. A standard 12‑lead ECG was obtained at baseline (upon hospital admission) and again at 90 min post-PPCI. For each patient, the sum of ST-segment elevations was measured at 20 milliseconds after the J-point across all infarct-related leads. STR was calculated using the following formula:

$$\mathrm{STR}=\left(\frac{\mathrm{ST}-\text{segment}\;\mathrm{sum}\;\mathrm{at}\;90\;\min}{\mathrm{ST}-\text{segment}\;\mathrm{sum}\;\mathrm{at}\;\text{baseline}\;}-1\right)\times 100$$

Patients were categorized based on the degree of ST-segment resolution:

• Complete STR: ≥70 % resolution
• Partial STR: 30–70 % resolution
• Incomplete STR: <30 % resolution

For this study, STR ≥70 % was considered indicative of successful myocardial reperfusion.

To evaluate functional recovery, the improvement in LVEF was determined by calculating the difference between the post-procedure and baseline LVEF values. LVEF was assessed using transthoracic echocardiography, with the Biplane Simpson's method applied for calculations by a board-certified cardiologist. Echocardiographic evaluations were performed at two intervals: before the intervention and 40 days after the intervention.

Finally, MACE was defined as a combination of cardiovascular death, non-fatal myocardial infarction, stroke, and any form of revascularization at 40 days. These outcomes were defined based on the criteria established by the Academic Research Consortium (ARC).

### 2.4 Sample size calculation

This study was designed to assess the effect of adenosine on the incidence of the no-reflow phenomenon, with the primary endpoint defined as post-PCI TIMI flow grade. The required sample size was calculated using a two-tailed test, assuming a 95 % confidence level and 90 % power to detect a minimum clinically meaningful difference of 30 % in the occurrence of TIMI flow grade <3, based on the study by Naghash-Tabrizi et al. [11].

Although the primary outcome was assessed immediately after PCI and not subject to loss to follow-up, the sample size was conservatively inflated to account for potential attrition related to the 40-day follow-up period for secondary endpoints, including changes in left ventricular ejection fraction (LVEF) and major adverse cardiac events (MACE). Based on this consideration, an initial sample size of at least 134 patients was estimated, incorporating a 20 % anticipated dropout rate. To further strengthen the statistical power and ensure robustness across all outcomes, a total of 250 patients were ultimately enrolled.

1−α=0.95→Z1−α2=Z0.975=1.96

$$1-\beta =0.90\to Z_{1-\beta }=Z_{0.90}=1.28$$

**Figure:**
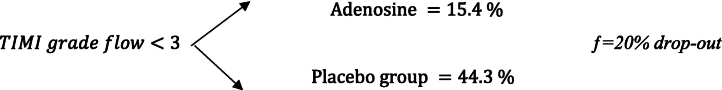

n=Z1−α2+Z1−β2×P11−P1+P21−P2P1−P22×11−f

$$n=\frac{{\left(1.96+1.28\right)}^{2}??\left[0.154\left(1-0.154\right)+0.443\left(1-0.443\right)\right]}{{\left(0.154-0.443\right)}^{2}}\times \frac{1}{1-0.2}$$

$$=106\;\text{patients}\;\mathrm{per}\;\text{group}\;\left(\text{total}\;134\right)$$

### 2.5 Randomization and allocation

Randomization was performed using a block randomization method (25 blocks of 10 patients each) via Sealedenvelope.com. Within each block, 5 patients were assigned to each group to ensure balance. Group assignments were placed in sequentially numbered, opaque, sealed envelopes and opened only at enrollment. The allocation process was managed by a staff member independent of patient care and data collection.

### 2.6 Ethic statement

The study protocol was approved by the Ethics Committee of Guilan University of Medical Sciences (IR.GUMS.REC.1402.108) and adhered to the principles of the Declaration of Helsinki [12]. Written informed consent was obtained from all participants. The trial was registered on the Iranian Clinical Trials Registry (IRCT20220809055645N6) at www.irct.behdasht.gov.ir.

### 2.7 Statistical analysis

All statistical analyses were performed using IBM SPSS Statistics version 27.0.1. Categorical variables were summarized as frequencies and percentages, and continuous variables were expressed as mean ± standard deviation. The normality of continuous variables was assessed using the Shapiro-Wilk test, supported by visual inspection of histograms and Q-Q plots. Categorical variables were compared using the Chi-square test, while Continuous variables were compared using the independent samples *t*-test for normally distributed data and the Mann-Whitney *U* test for non-normally distributed data. Multivariable logistic and linear regression were used to account for differences in baseline clinical and procedural characteristics for primary and secondary outcomes. All statistical tests were two-tailed, and a *p*-value of less than 0.05 was considered to indicate statistical significance.

## 3 Results

### 3.1 Baseline characteristics

A total of 240 patients were randomized equally to the adenosine (*n* = 120) and placebo (n = 120) groups. Baseline demographic, clinical, and procedural characteristics are summarized in Table 1.

**Table 1:** Comparison of baseline clinical and procedural characteristics between the adenosine and placebo groups. This table summarizes demographic, clinical, and procedural characteristics of patients randomized to receive either adenosine or a placebo. Data are presented as mean ± SD or n (%), as appropriate.

Basic characteristics	Adenosine (*N* = 120)	Placebo (N = 120)	p-Value
Age (years)	58.02 ± 14.31	58.37 ± 15.06	0.812^b^
Gender			0.123^c^
Female	42 (35 %)	31 (25.8 %)	
Male	78 (65 %)	89 (74.2 %)	
Diabetes	64 (53.3 %)	46 (38.3 %)	0.02^c^
Dyslipidemia	72 (60 %)	49 (40.8 %)	0.003^c^
Hypertension	54 (45 %)	52 (43.3 %)	0.795^c^
Smoking	41 (34.2 %)	58 (48.3 %)	0.026^c^
GFR (ml/min)	80.46 ± 18.61	75.53 ± 19.49	0.04^b^
Artery			0.846^c^
RCA	36 (30 %)	35 (29.2 %)	
LCX	14 (11.7 %)	17 (14.2 %)	
LAD	70 (58.3 %)	68 (56.7 %)	
Door-to-balloon time			0.734^c^
<120 min	38 (31.7 %)	41 (34.2 %)	
120–360 min	56 (46.7 %)	50 (41.7 %)	
>360 min	26 (21.7 %)	29 (24.2 %)	
Thrombus burden			0.141^a^
Grade 0	0	0	
Grade 1	1 (0.8 %)	0	
Grade 2	0	0	
Grade 3	10 (8.3 %)	21 (17.5 %)	
Grade 4	41 (34.2 %)	39 (32.5 %)	
Grade 5	68 (56.7 %)	60 (50 %)	
Balloon pre-dilatation	41 (34.2 %)	44 (36.7 %)	0.686^c^
Balloon post-dilatation	57 (47.5 %)	51 (42.5 %)	0.436^c^
Glycoprotein IIb/IIIa use	31 (25.8 %)	33 (27.5 %)	0.77^c^
Thrombus suction	20 (16.7 %)	10 (8.3 %)	0.051^c^
Initial TIMI flow grade			0.018^c^
0	86 (71.7 %)	65 (54.2 %)	
1	26 (21.7 %)	44 (36.7 %)	
2	8 (6.7 %)	11 (9.2 %)	
Initial TIMI frame count	20.54 ± 6.95	20.43 ± 6.69	0.973^b^
Initial LVEF (%)	33.21 ± 7.98	32.71 ± 8.22	0.63^b^

Abbreviations: GFR, glomerular filtration rate; RCA, right coronary artery; LCX, left circumflex artery; LAD, left anterior descending artery; TIMI, Thrombolysis in Myocardial Infarction; LVEF, left ventricular ejection fraction.

a Fisher's Exact Test.

b Mann Whitney *U* Test.

c Chi Square Test.

The two groups were well matched in terms of age (58.0 ± 14.3 vs. 58.4 ± 15.1 years, *p* = 0.812) and sex distribution (female: 35 % vs. 25.8 %, *p* = 0.123). The prevalence of hypertension was similar between groups (45.0 % vs. 43.3 %, *p* = 0.795).

However, significant differences were observed in several cardiovascular risk factors. Diabetes mellitus was more frequent in the adenosine group compared with placebo (53.3 % vs. 38.3 %, *p* = 0.020), as was dyslipidemia (60.0 % vs. 40.8 %, *p* = 0.003). Conversely, current smoking was more common in the placebo group (48.3 % vs. 34.2 %, *p* = 0.026). Baseline renal function was slightly higher in the adenosine group (GFR: 80.5 ± 18.6 vs. 75.5 ± 19.5 mL/min, *p* = 0.040).

Procedural characteristics, including culprit artery distribution, door-to-balloon time, use of pre- or post-dilatation, glycoprotein IIb/IIIa inhibitors, and thrombus suction, did not differ significantly between groups (*p* > 0.05 for all).

Regarding angiographic parameters, initial TIMI flow grade showed a significant difference between groups (*p* = 0.018). A higher proportion of patients in the adenosine group presented with TIMI 0 flow (71.7 % vs. 54.2 %), whereas TIMI 1 flow was more frequent in the placebo group (36.7 % vs. 21.7 %). Initial TIMI frame count (20.5 ± 7.0 vs. 20.4 ± 6.7, *p* = 0.973) and baseline LVEF (33.2 ± 8.0 % vs. 32.7 ± 8.2 %, *p* = 0.630) were comparable between groups.

### 3.2 Clinical outcomes

Clinical outcomes at follow-up are summarized in Table 2. Final angiographic success, as assessed by TIMI flow grade 3, was achieved in the majority of patients in both groups (85.0 % vs. 80.8 %, *p* = 0.738). TIMI frame count tended to be lower in the adenosine group compared with placebo (13.5 ± 7.2 vs. 15.0 ± 8.1), although this difference did not reach statistical significance (*p* = 0.089).

**Table 2:** Comparison of clinical outcomes between the adenosine and placebo groups. Outcomes include TIMI flow grade, ST-segment resolution, ejection fraction after 40 days, no-reflow incidence, and major adverse cardiac events. Data are presented as mean ± SD or n (%).

Outcome	Adenosine (*N* = 120)	Placebo (N = 120)	*p*-Value
TIMI flow grade			0.738^a^
0	1 (0.8 %)	3 (2.5 %)	
1	6 (5 %)	7 (5.8 %)	
2	11 (9.2 %)	13 (10.8 %)	
3	102 (85 %)	97 (80.8 %)	
TIMI frame count	13.5 ± 7.17	14.96 ± 8.06	0.089^b^
STR			0.131^c^
<30 %	6 (5 %)	10 (8.3 %)	
30–70 %	58 (48.3 %)	69 (57.5 %)	
≥70 %	56 (46.7 %)	41 (34.2 %)	
LVEF after 40 days (%)	46.61 ± 9.27	44.38 ± 10.68	0.086^b^
MACE	5 (4.2 %)	3 (2.5 %)	0.72^a^
No-reflow based on TIMI flow grade	18 (15 %)	23 (19.2 %)	0.391^c^
No-reflow based on TIMI frame count	8 (6.7 %)	8 (6.7 %)	1.00^c^
Change in LVEF (%)	**13.83 ± 7.43**	**12.08 ± 8.39**	**0.043**^b^
Relative changes in LVEF	**0.47 ± 0.42**	**0.40 ± 0.34**	0.102^b^

Abbreviations: TIMI, Thrombolysis in Myocardial Infarction; STR, ST-segment resolution; LVEF, left ventricular ejection fraction; MACE, major adverse cardiac events.

Statistically significant values (*p* < 0.05) are in bold.

a Fisher's Exact Test.

b Mann Whitney *U* Test.

c Chi Square Test.

Electrocardiographic assessment showed greater ST-segment resolution of≥70 % in the adenosine group compared with the placebo group (46.7 % vs. 34.2 %), although this difference was not statistically significant (*p* = 0.131).

Left ventricular systolic function at 40 days was numerically higher in patients receiving adenosine (46.6 ± 9.3 % vs. 44.4 ± 10.7 %, *p* = 0.086). Importantly, the absolute change in LVEF from baseline was significantly greater in the adenosine group compared with placebo (13.8 ± 7.4 % vs. 12.1 ± 8.4 %, *p* = 0.043). Relative change in LVEF did not differ significantly between groups (0.47 ± 0.42 vs. 0.40 ± 0.34, *p* = 0.102).

The incidence of no-reflow, whether defined by TIMI flow grade (15.0 % vs. 19.2 %, *p* = 0.391) or TIMI frame count (6.7 % vs. 6.7 %, *p* = 1.00), was comparable between groups. Similarly, rates of major adverse cardiac events (MACE) were low and not statistically different (4.2 % vs. 2.5 %, *p* = 0.720).

### 3.3 ANCOVA for LVEF at 40 days

An ANCOVA model was performed to examine the association between treatment group and LVEF at 40 days, adjusting for baseline LVEF and other clinical covariates (Table 3). The analysis demonstrated that group assignment was an independent predictor of follow-up LVEF. After adjusting for covariates, patients in the adenosine group had a significantly higher mean LVEF at 40 days compared with placebo (mean difference: +9.73 units, *p* = 0.020; 95 % CI: −17.9 to −1.55).

**Table 3:** ANCOVA examining the association between study groups and LVEF at 40 days, adjusting for baseline LVEF and other covariates.

	Coefficient	Std. error	t	*p*-Value	95 % CI	Eta square	Observed power
Intercept	22.46	3.86	5.81	<0.001	[14.85, 30.07]	0.127	1
Group; Control	−9.73	4.15	−2.34	**0.02**	[−17.9, −1.55]	0.02	0.65
LVEF1	0.6	0.09	6.75	**<0.001**	[0.43, 0.78]	0.16	1
Group (control) *LVEF1	0.25	0.12	2.06	**0.04**	[0.01, 0.48]	0.018	0.53
DM	−1.69	1.05	−1.61	0.11	[−3.77, 0.38]	0.011	0.36
DLP	−0.35	1.04	−0.34	0.74	[−2.39, 1.69]	0.000	0.63
Smoker	−2.31	1.02	−2.28	**0.024**	[−4.31, −0.31]	0.022	0.62
GFR	0.075	0.026	2.88	**0.004**	[0.024, 0.125]	0.035	0.82

When baseline LVEF is held constant, mean LVEF at 40 days is 9.73 units higher in the intervention group than in the control group (p = 0.02). The Group × baseline LVEF interaction is significant (p = 0.04), indicating that the assumption of homogeneity of slopes is violated; for every unit increase in baseline LVEF, the difference in adjusted mean LVEF40 between intervention and control groups changes by 0.25 units. Smoking status (p = 0.024) and GFR (p = 0.004) were also significantly associated with LVEF at 40 days. Bold p-values indicate statistically significant associations (p < 0.05).

Baseline LVEF was strongly associated with follow-up LVEF (β = 0.60, *p* < 0.001), confirming its role as the most influential covariate. Importantly, the group × baseline LVEF interaction was statistically significant (*p* = 0.040), indicating violation of the homogeneity of slopes assumption. This interaction suggests that for each 1-unit increase in baseline LVEF, the difference in adjusted mean LVEF at 40 days between groups changed by 0.25 units, with the slope in the control group being steeper.

Among additional covariates, smoking status was negatively associated with follow-up LVEF (β = −2.31, *p* = 0.024), and higher baseline GFR was positively associated with LVEF at 40 days (β = 0.075, *p* = 0.004). Diabetes and dyslipidemia were not significantly related to the outcome.

### 3.4 Subgroup analysis by thrombus burden

Clinical outcomes stratified by thrombus burden are shown in Table 4. Among patients who underwent thrombus aspiration (*n* = 30), the incidence of no-reflow based on TIMI flow grade (45 % vs. 70 %, *p* = 0.26) or TIMI frame count (20 % vs. 50 %, *p* = 0.12) did not differ significantly between the adenosine and placebo groups. Changes in LVEF (8.0 ± 8.6 % vs. 4.0 ± 11.5 %, *p* = 0.15) and relative changes in LVEF (0.30 ± 0.39 vs. 0.15 ± 0.49, *p* = 0.13) were numerically greater in the adenosine group but did not reach statistical significance.

**Table 4:** Comparison of no-reflow phenomenon and left ventricular functional recovery between adenosine and placebo groups stratified by thrombus burden.

		With thrombus; n = 30			Without thrombus; n = 210		
		Adenosine	Placebo	p-Value	Adenosine	Placebo	p-Value
NRP based on TIMI flow grade	Yes	9 (45 %)	7 (70 %)	0.26^a^	9 (9 %)	16 (14.5 %)	0.21^b^
	No	11 (55 %)	3 (30 %)		91 (91 %)	94 (85.5 %)	
NRP based on TIMI frame count	Yes	4 (20 %)	5 (50 %)	0.12^a^	4 (4 %)	3 (2.7 %)	0.71^a^
	No	16 (80 %)	5 (50 %)		96 (96 %)	107 (97.3 %)	
Changes in LVEF; mean ± SD		8 ± 8.64	4 ± 11.49	0.15^c^	14.99 ± 6.62	12.82 ± 7.71	**0.012**^c^
Relative changes in LVEF; mean ± SD		0.3 ± 0.39	0.15 ± 0.49	0.13^c^	0.51 ± 0.42	0.42 ± 0.31	**0.039**^c^

Bold p-values indicate statistically significant differences between groups (p < 0.05). In this table, the changes in absolute LVEF (p = 0.012) and relative LVEF (p = 0.039) among patients without thrombus were significantly greater in the adenosine group compared with placebo, reflecting improved left ventricular functional recovery.

a Fisher's exact test.

b Chi-square test.

c Mann Whitney *U* test.

In contrast, among patients without thrombus aspiration (*n* = 210), no-reflow rates were comparable between groups, whether defined by TIMI flow grade (9.0 % vs. 14.5 %, *p* = 0.21) or TIMI frame count (4.0 % vs. 2.7 %, *p* = 0.71). However, both absolute and relative improvements in LVEF were significantly greater in the adenosine group. The mean change in LVEF was 14.99 ± 6.6 % in the adenosine group compared with 12.82 ± 7.7 % in the placebo group (*p* = 0.012). Similarly, relative LVEF improvement was higher in patients treated with adenosine (0.51 ± 0.42 vs. 0.42 ± 0.31, *p* = 0.039).

### 3.5 Predictors of no-reflow phenomenon

Logistic regression analysis was performed to identify independent predictors of the no-reflow phenomenon as defined by TIMI flow grade (Table 5). Treatment with adenosine was not significantly associated with no-reflow (OR: 0.69, 95 % CI: 0.33–1.46, *p* = 0.332).

**Table 5:** Logistic regression analysis of clinical factors associated with the no-reflow phenomenon based on TIMI flow grade. Data include regression coefficients (B), standard errors, Wald statistic, degrees of freedom (df), *p*-values, odds ratios (Exp(B)), and 95 % confidence intervals.

Variable	B	S.E.	Wald	p-Value	OR	95 % C.I. for OR	
						Lower	Upper
Group (adenosine)	−0.371	0.383	0.940	0.332	0.690	0.326	1.461
DM (yes)	1.313	0.406	10.481	**0.001**	3.718	1.679	8.232
DLP (yes)	0.166	0.384	0.187	0.665	1.181	0.557	2.504
Smoke (yes)	0.805	0.374	4.644	**0.031**	2.236	1.076	4.650
eGFR	−0.025	0.009	7.334	**0.007**	0.976	0.958	0.993
Constant	−0.738	0.764	0.935	0.333	0.478		

Bold p-values indicate statistically significant associations (p < 0.05). In this analysis, diabetes mellitus (p = 0.001), smoking (p = 0.031), and eGFR (p = 0.007) were significantly associated with the no-reflow phenomenon, reflecting meaningful effects on the odds of occurrence.

Abbreviations: DM: Diabetes mellitus, DLP: Dyslipidemia.

Among clinical covariates, diabetes mellitus emerged as a strong predictor of no-reflow (OR: 3.72, 95 % CI: 1.68–8.23, *p* = 0.001). Smoking was also significantly associated with increased risk (OR: 2.24, 95 % CI: 1.08–4.65, *p* = 0.031). Baseline renal function, as measured by eGFR, was inversely related to no-reflow (OR per unit increase: 0.98, 95 % CI: 0.96–0.99, *p* = 0.007). Dyslipidemia did not demonstrate a significant association (OR: 1.18, 95 % CI: 0.56–2.50, *p* = 0.665).

### 3.6 Predictors of no-reflow phenomenon based on TIMI frame count

Logistic regression analysis of predictors of no-reflow as defined by TIMI frame count, is presented in Table 6. It should be noted that the total number of no-reflow events was limited (*n* = 14), which constrains the stability of the model when adjusting for multiple covariates.

**Table 6:** Logistic regression analysis of clinical factors associated with the no-reflow phenomenon based on TIMI frame count. Data include regression coefficients, standard errors, Wald statistics, p-values, odds ratios (Exp(B)), and 95 % CIs.

Variables	B	S.E.	Wald	p-Value	OR	95 % C.I. for OR	
						Lower	Upper
Group (adenisine)	0.051	0.562	0.008	0.928	1.052	0.349	3.169
DM (yes)	1.351	0.628	4.624	**0.032**	3.861	1.127	13.228
DLP (yes)	−0.135	0.589	0.053	0.819	0.874	0.276	2.770
Smoke (yes)	1.803	0.615	8.593	**0.003**	6.070	1.818	20.267
eGFR	−0.010	0.013	0.606	0.436	0.990	0.965	1.016
Constant	−3.613	1.233	8.587	**0.003**	0.027		

Abbreviations: DM: Diabetes mellitus, DLP: Dyslipidemia.

Bold p-values indicate statistically significant associations (p < 0.05). **Note:** The number of no-reflow events based on TIMI frame count (n = 14) is small, which limits the reliability of the logistic regression model with five variables.

Adenosine treatment was not significantly associated with the occurrence of no-reflow (OR: 1.05, 95 % CI: 0.35–3.17, *p* = 0.928). Among clinical covariates, diabetes mellitus was independently associated with a higher risk of no-reflow (OR: 3.86, 95 % CI: 1.13–13.23, *p* = 0.032), and smoking was a strong predictor (OR: 6.07, 95 % CI: 1.82–20.27, *p* = 0.003). Dyslipidemia (OR: 0.87, *p* = 0.819) and baseline renal function as measured by eGFR (OR per unit increase: 0.99, *p* = 0.436) were not significantly associated with no-reflow.

### 3.7 Baseline characteristics and propensity score matching

Propensity score matching (nearest neighbor, caliper = 0.2, ratio = 1, without replacement) was applied to balance key covariates (Diabetes, Dyslipidemia, Smoking) between the treatment and control groups. After matching, baseline characteristics were well balanced, minimizing confounding in subsequent analyses.

### 3.8 No-reflow phenomenon (NRP) based on TIMI grade

Logistic regression analysis showed no statistically significant association between treatment and NRP in either the matched or the original dataset. In the matched data, the odds ratio (OR) was 0.74 (95 % CI, 0.34–1.58; *p* = 0.44), while in the original data, the OR was 0.63 (95 % CI, 0.30–1.30; *p* = 0.21). The difference in ORs between matched and raw data was minimal (Diff. OR = −0.11) (Table 7).

**Table 7:** Logistic regression results for no-reflow phenomenon (NRP) based on TIMI grade, including rosenbaum sensitivity analysis.

	OR	Std. error	Statistics	p. value	95 % CI for OR		Diff. OR
					Low	High	
Treatment in matched data	0.74	0.39	−0.78	0.44	0.34	1.58	−0.11
Treatment in raw data	0.63	0.37	−1.24	0.21	0.3	1.3	

As shown in the table above, the p-value for the association between treatment and the outcome event is not statistically significant in either dataset.

Based on the Rosenbaum sensitivity test, there is no difference in the results between the matched data and the original data.

Rosenbaum bounds value of Γ = 1.

Rosenbaum sensitivity analysis indicated no difference in results between matched and original data (Γ = 1), suggesting that the findings are robust to unmeasured confounding.

### 3.9 No-reflow phenomenon (NRP) based on TIMI count

Similarly, for NRP defined by TIMI count, logistic regression revealed no significant treatment effect. In matched data, OR was 1.40 (95 % CI, 0.45–4.59; *p* = 0.56), and in raw data, OR was 1.00 (95 % CI, 0.33–3.00; *p* = 0.99), with a Diff. OR of −0.4.

Rosenbaum sensitivity analysis again showed Γ = 1, indicating that the results are not sensitive to hidden bias and are consistent across matched and original datasets.

Overall, treatment was not significantly associated with the occurrence of NRP, and the results were robust to potential unmeasured confounding as assessed by Rosenbaum sensitivity analysis (Table 8).

**Table 8:** Logistic regression results for no-reflow phenomenon (NRP) Based on TIMI count, including rosenbaum sensitivity analysis.

	OR	Std. error	Statistics	p. value	95 % CI for OR		Diff. OR
					Low	High	
Treatment in matched data	1.4	0.58	0.58	0.56	0.45	4.59	−0.4
Treatment in raw data	1	0.55	−0.001	0.99	0.33	3	

Based on the Rosenbaum sensitivity test, there is no difference in the results between the matched data and the original data.

Rosenbaum bounds value of Γ = 1.

Intracoronary adenosine was well tolerated. No clinically significant bradyarrhythmia, hypotension, or bronchospasm occurred. Minor, transient effects, such as brief flushing or mild hypotension, were observed in a few patients but resolved spontaneously without intervention.

## 4 Discussion

In this randomized, triple-blind, placebo-controlled trial, we evaluated the effect of prophylactic intracoronary adenosine administered prior to stent implantation on the incidence of no-reflow and myocardial recovery. Although adenosine did not significantly reduce no-reflow compared with placebo, it was consistently associated with greater improvements in left ventricular ejection fraction (LVEF) at 40 days, supporting a cardioprotective effect that extends beyond immediate angiographic outcomes.

Adenosine's pharmacologic profile, including vasodilatory, anti-inflammatory, and antiplatelet properties mediated via A2 receptor activation, provides a strong mechanistic rationale for its use in primary PCI [4]. Prior studies have yielded conflicting results: early trials such as AMISTAD and AMISTAD-II suggested infarct size reduction with intravenous adenosine, whereas later investigations reported more modest or inconsistent benefits [13,14]. The REOPEN-AMI trial [15] demonstrated favorable ventricular remodeling with intracoronary administration, yet the broader evidence base has been limited by heterogeneity in dosing strategies, timing, and delivery techniques, as well as by concerns regarding adverse effects such as bradycardia, hypotension, and bronchospasm.

In our cohort, adenosine did not significantly alter the angiographic incidence of no-reflow, regardless of whether it was defined by TIMI flow grade or frame count. However, subgroup and adjusted analyses consistently demonstrated that adenosine enhanced myocardial functional recovery. Importantly, improvements in absolute and relative LVEF were evident even among patients who did not undergo thrombus aspiration, indicating a pharmacologic effect independent of procedural adjuncts. These findings align with the work of Zhang et al. [16], who observed improvements in TIMI flow, myocardial blush grade, ST-segment resolution, and LVEF, particularly with higher doses of adenosine. Similarly, Ahmed Hatata et al. [17] reported enhanced myocardial salvage on cardiac magnetic resonance imaging (MRI) at three months and a significant reduction in microvascular obstruction (MVO) at 48 h post-PCI, despite the absence of immediate angiographic improvements. These findings underscore the potential for adenosine to exert beneficial microvascular effects, which may not always be evident in early angiographic assessments.

Together, these results suggest that angiography alone may underestimate adenosine's therapeutic impact and that functional and imaging endpoints are essential for assessing microvascular recovery.

Baseline imbalances in key risk factors may have obscured the apparent effect of adenosine on angiographic no-reflow. The adenosine group had a higher prevalence of diabetes and dyslipidemia, conditions strongly associated with microvascular dysfunction, endothelial impairment, and reduced responsiveness to vasodilators. These comorbidities likely blunted the protective effects of adenosine, making angiographic differences less apparent despite a true biologic benefit. Together, these opposing baseline characteristics, combined with the limited sample size, may have narrowed observable differences in angiographic indices, even though functional outcomes, such as LVEF recovery at 40 days, clearly demonstrated a benefit of adenosine. In the present study, we applied propensity score matching to account for potential confounding by baseline characteristics such as diabetes, dyslipidemia, and smoking. Logistic regression analyses performed on both the matched and original datasets demonstrated no significant association between intracoronary adenosine treatment and the occurrence of the no-reflow phenomenon, regardless of whether NRP was defined by TIMI grade or TIMI count.

To further assess the robustness of our findings, we conducted Rosenbaum sensitivity analysis. The Γ value of 1 indicates that the observed non-significant associations are unlikely to be affected by unmeasured confounding. Together, these analyses suggest that our results are stable and not driven by baseline imbalances or hidden bias.

A key procedural consideration is the site of drug delivery. In our study, adenosine was administered proximally through the guiding catheter, a method that may limit local efficacy due to rapid dilution and metabolism. Recent work, such as that of Abu Arab et al. [18], highlights the superiority of distal coronary bed delivery, which achieves higher local concentrations, minimizes systemic side effects, and may mechanically aid thrombus clearance. Despite promising results from limited case reports, distal administration remains underutilized. Given the potential benefits, incorporating distal adenosine delivery should be considered as part of the standard treatment algorithm for refractory no-reflow [19].

Prospective randomized trials are needed to definitively establish the safety and efficacy of distal versus proximal adenosine administration and to develop standardized protocols for clinical use.

Our findings also underscore the broader principle that pharmacologic therapy alone may be insufficient to optimize reperfusion outcomes in STEMI. Prior work by Sadeghian [9] and Naghshtabrizi [11] demonstrated improvements in angiographic indices of perfusion with adenosine, but without translation into major adverse cardiac events (MACE) or sustained LVEF benefit. Similarly, in our study, adenosine's effect on angiographic no-reflow was neutral, yet functional recovery was enhanced. This discrepancy reinforces the complex interplay between pharmacologic and mechanical factors in myocardial reperfusion and highlights the need for integrative treatment strategies.

Taken together, our data suggest that adenosine confers a measurable improvement in post-PCI myocardial recovery, even in the absence of clear angiographic benefit. The borderline associations we observed with both EF recovery and no-reflow incidence point to a biologic signal that may reach clinical significance with optimized patient selection, distal delivery, and tailored dosing strategies. Although adenosine is unlikely to serve as a stand-alone solution for no-reflow, it may play an important role as part of a multimodal reperfusion strategy aimed at improving microvascular and functional outcomes in STEMI.

### 4.1 Future directions

Future research should aim to optimize intracoronary adenosine administration to enhance microvascular protection and functional recovery. Distal delivery, higher or tailored dosing, and precise timing may improve myocardial salvage while minimizing systemic side effects. Stratifying patients by baseline microvascular risk factors, such as diabetes or dyslipidemia, could help identify subgroups most likely to benefit. Incorporating advanced imaging and longer-term functional endpoints, as well as evaluating adenosine in combination with optimized mechanical reperfusion strategies, will be important to clarify its role as an adjunctive therapy in STEMI management.

### 4.2 Limitations

1. Lack of Statistical Significance: Although adenosine showed promising trends, the lack of significant differences in key clinical outcomes, such as no-reflow and LVEF recovery, limits the strength of the conclusions.
2. Patient Population Variability: While adjustments were made for confounders, variations in patient characteristics (e.g., diabetes, comorbidities) may still have influenced the results.
3. Method of Drug Delivery: The standard proximal delivery method used in the study may have reduced adenosine's effectiveness compared to other techniques, such as distal coronary bed injection.
4. Potential for Unmeasured Confounders: Despite adjustments, other unmeasured variables (e.g., medication adherence, subtle procedural differences) may have affected the outcomes.
5. Although TIMI flow grades and corrected TIMI frame counts were assessed by a single experienced interventional cardiologist to ensure consistency, the lack of formal intra- and inter-observer variability assessment represents a potential source of observer bias. This may have influenced the objectivity of angiographic interpretations.
6. Short Follow-Up Duration: The study primarily assessed short-term outcomes, and the long-term impact of adenosine on clinical outcomes like MACE and mortality remains unclear.

## 5 Conclusion

In this randomized, triple-blind trial, prophylactic intracoronary adenosine prior to stent implantation did not significantly reduce angiographic no-reflow in STEMI patients undergoing primary PCI, likely due to baseline imbalances in key risk factors such as diabetes and dyslipidemia. However, adenosine was consistently associated with greater left ventricular functional recovery at 40 days, including in patients who did not undergo thrombus aspiration, indicating a direct cardioprotective effect on the microvasculature and myocardial tissue. These findings suggest that while adenosine may have a limited impact on immediate angiographic outcomes, it can enhance myocardial salvage and functional recovery, supporting its potential role as an adjunctive therapy in STEMI management. Further studies are warranted to optimize delivery techniques, dosing, and patient selection to maximize its clinical benefit.

## CRediT authorship contribution statement

**Fatemeh Baharvand:** Writing – original draft, Investigation, Data curation, Conceptualization. **Mohammadreza Aghajankhah:** Writing – review & editing, Validation, Methodology, Formal analysis. **Shiva Parvaneh:** Writing – review & editing, Supervision. **Bahareh Gholami Chaboki:** Writing – review & editing. **Francesca Maria Di Muro:** Writing – review & editing.

## Ethical approval

The study protocol was approved by the Ethics Committee of Guilan University of Medical Sciences (IR.GUMS.REC.1402.108).

## Ethical statement

This study was conducted in accordance with the principles outlined in the Declaration of Helsinki. The study protocol was reviewed and approved by the Ethics Committee of Guilan University of Medical Sciences (Approval ID: IR.GUMS.REC.1402.108). Written informed consent was obtained from all participants prior to their enrollment in the study. All procedures were performed in accordance with institutional and national research committee standards and adhered to ethical principles governing clinical research involving human subjects. Furthermore, the trial was prospectively registered in the Iranian Registry of Clinical Trials (IRCT) under the registration number IRCT20220809055645N6.

## Funding

This research did not receive any specific grant from funding agencies in the public, commercial, or not-for-profit sectors.

## Declaration of competing interest

The authors declare no conflicts of interest.
